# Anti-TNF-α Agent Tamarind Kunitz Trypsin Inhibitor Improves Lipid Profile of *Wistar* Rats Presenting Dyslipidemia and Diet-induced Obesity Regardless of *PPAR-γ* Induction

**DOI:** 10.3390/nu11030512

**Published:** 2019-02-27

**Authors:** Fabiana M. C. Carvalho, Vanessa C. O. Lima, Izael S. Costa, Anna B. S. Luz, Fernando V. L. Ladd, Alexandre C. Serquiz, Raul H. Bortolin, Vivian N. Silbiger, Bruna L. L. Maciel, Elizeu A. Santos, Ana H. A. Morais

**Affiliations:** 1 Biochemistry Postgraduate Program, Biosciences Center, Federal University of Rio Grande do Norte, Natal, RN 59078-970, Brazil; fabicoimbra@hotmail.com (F.M.C.C.); vanessalima_nutri@yahoo.com.br (V.C.O.L.); izaelsousa@hotmail.com (I.S.C.); elizeu.ufrn@gmail.com (E.A.S.); 2 Nutrition Postgraduate Program, Center for Health Sciences, Federal University of Rio Grande do Norte, Natal, RN 59078-970, Brazil; abeatrizluz@gmail.com (A.B.S.L.); viviansilbiger@hotmail.com (V.N.S); brunalimamaciel@gmail.com (B.L.L.M.); 3 Department of Morphology, Biosciences Center, Federal University of Rio Grande do Norte, Natal, RN 59078-970, Brazil; fernandoladd@gmail.com; 4 Course of Nutrition, Center University of Rio Grande do Norte, Natal, RN 59014-545, Brazil; alexandreserquiz@gmail.com; 5 Pharmaceutical Sciences Post Graduate Program, Center for Health Sciences, Federal University of Rio Grande do Norte, Natal, RN 59078-970, Brazil; raulhbortolin@yahoo.com.br; 6 Department of Clinical and Toxicological Analysis, Center for Health Sciences, Federal University of Rio Grande do Norte, Natal, RN 59078-970, Brazil; 7 Department of Nutrition, Center for Health Sciences, Federal University of Rio Grande do Norte, Natal, RN 59078-970, Brazil; 8 Department of Biochemistry, Center for Biosciences, Federal University of Rio Grande do Norte, Natal, RN 59078-970, Brazil

**Keywords:** inflammation, *Tamarindus indica* L., triglycerides, VLDL

## Abstract

The increasing prevalence of obesity and, consequently, chronic inflammation and its complications has increased the search for new treatment methods. The effect of the purified tamarind seed trypsin inhibitor (TTIp) on metabolic alterations in *Wistar* rats with obesity and dyslipidemia was evaluated. Three groups of animals with obesity and dyslipidemia were formed, consuming a high glycemic index and glycemic load (HGLI) diet, for 10 days: Obese/HGLI diet; Obese/standard diet; Obese/HGLI diet + TTIp (730 μg/kg); and one eutrophic group of animals was fed a standard diet. Rats were evaluated daily for food intake and weight gain. On the 11th day, animals were anesthetized and sacrificed for blood and visceral adipose tissue collection. TTIp treated animals presented significantly lower food intake than the untreated group (*p* = 0.0065), TG (76.20 ± 18.73 mg/dL) and VLDL-C (15.24 ± 3.75 mg/dL). Plasma concentrations and *TNF-α* mRNA expression in visceral adipose tissue also decreased in obese animals treated with TTIp (*p* < 0.05 and *p* = 0.025, respectively) with a negative immunostaining. We conclude that TTIp presented anti-TNF-α activity and an improved lipid profile of *Wistar* rats with dyslipidemia and obesity induced by a high glycemic index and load diet regardless of *PPAR-γ* induction.

## 1. Introduction 

There are indications that hypolipidic and hyperglycemic diets considerably stimulate lipogenesis [1], increasing the expression of lipogenic enzymes [2] by means of transcription factors, such as sterol regulatory binding proteins (SREBP) [3] and activated carbohydrate responsive element-binding protein (ChREBP), which is activated in response to high glycemia and stimulation of the nuclear receptor peroxisome proliferator-activated receptor-gamma (PPAR-γ) [4].

According to Virdis et al. [5], the hyperglycemic diet is combined with risk factors for dyslipidemia and obesity, similarly to lipid-rich diets. Possibly, this relationship is attributed to the higher stimulus to hepatic lipogenesis, especially in the synthesis of triglycerides and consequently the very low density lipoproteins (VLDL-C), through a greater supply of plasma glucose.

Obesity is defined as concentrated or generalized fatty acid deposition, derived from nutritional imbalance associated or not with genetic or endocrine metabolic disorders [6]. It is an important risk to type 2 diabetes mellitus, arterial hypertension, coronary artery disease, dyslipidemias, and certain types of cancer and circulatory disorders [7,8]. It is a complex chronic disease in which adipose tissue is infiltrated by activated macrophages and releases excessive amounts of inflammatory cytokines, such as tumor necrosis factor-α (TNF-α), plasminogen activator inhibitor 1 (PAI-1), interleukin-6 (IL-6), retinol-binding protein 4, macrophages chemoattractant protein 1 (MCP-1), and acute phase proteins [9]. These factors exert paracrine actions, which perpetuate local inflammation in the adipose tissue, and endocrine paracrine, which induces insulin resistance and vascular and cardiac dysfunctions [10].

Among the inflammatory factors, TNF-α is produced, not only by cells of the immune system, but also by cells of adipose tissue and possibly by other differentiated tissues [11]. In recent decades, a greater interest in TNF-α has been established because of its implication in the development of insulin resistance, its potential role as a regulator of adipose tissue mass, and its increased concentrations in the hypothalamus of animals submitted to hyperlipidic and hyperglycemic diet [12,13,14].

Fibrates and thiazolidinediones (TZDs) activate intracellular nuclear receptors such as PPARγ and TZDs, and reduce the expression of leptin and TNF-α [15,16], thereby reducing the inflammatory process by obesity. However, fibrates and TZDs cause some adverse and undesirable effects (hepatotoxicity) [15,16].

Also, there are drugs used for the reduction of inflammatory diseases such as rheumatoid arthritis, crohn’s disease, psoriasis, and ankylosing spondylitis. Among the biological agents approved for their treatment are those that act as antagonists of TNF-α, called anti-TNF-α [17,18]. Currently, five agents that block the action of TNF-α and are approved by FDA are available: etanercept (Enbrel^®^, Pfizer Ireland Pharmaceuticals, Dublin, Irland), infliximab (Remicade^®^, Cilag AG., Schaffhausen, Switzerland), adalimumab (Humira^®^, AbbVie Farmacêutica LTDA, Santo Amaro, São Paulo, Brazil), certolizumab-pegol (Cimzia^®^, Vetter Pharma-Fertigung GmbH & Co. KG, Langenargen, Germany), and golimumab (Simponi^®^, Baxter Pharmaceutical Solutions LLC, Bloomington, IN, USA) [19].

However, all these drugs cause alterations in the lipid profile, such as increased triglycerides, as well as the onset of type 2 diabetes and increased risk of atherosclerosis [20]. In this way, the search for bioactive substances from plants has been intensified in order to formulate new biopharmaceuticals.

In addition, pure molecules with inhibitory actions have been synthesized and used in several treatments [21,22]. As an example, orlistat reduces the digestion and/or absorption of nutrients [15]. Specific serotonin reuptake inhibitors (fluoxetine), as well as sibutramine, have been used in the treatment of obesity [22]. Herbal medicines, such as Potein^®^ (Dermo manipulações, São José dos Pinhais, Paraná, Brazil), composed of isolated trypsin inhibitors, have been used for the purpose of weight loss [23].

In this context, the isolation, purification, characterization, and bioavailability of trypsin inhibitors in seeds, among them tamarind, have been demonstrated in some studies [24,25,26]. In a study by our group, a partially purified trypsin inhibitor from tamarind seeds (TTI) [27] presented a satietogenic effect, reducing weight gain in eutrophic rats associated with the serum increase of cholecystokinin (CCK). In the study by Carvalho [28], besides the reduction of food consumption in animals with obesity and SM, the effect on TNF-α reduction and the lipid profile was observed for TTI. In addition, TTI did not present hepatotoxicity in the last two studies cited above [27,28].

We have also purified TTI (TTIp), and it presented an approximate mass of 18 kDa (TTIp), reducing leptin in obese mice [29]. With fully purified TTIp, it is relevant to investigate its action on satiety and biochemical parameters, as well as the effect on molecular markers altered by unbalanced, hyperglycemic diets. These will allow the comprehension of its mechanisms of action, stimulating studies of new potential drugs that may be effective for the prevention and/or control of diseases involving metabolic alterations.

## 2. Materials and Methods

### 2.1. Type of Study and Biological Material

This is an experimental study to investigate the effect of TTIp treatment on biochemical and molecular parameters administered for 10 days. The experiment was carried out with four groups of animals, according to the methodology described below.

### 2.2. In Vitro Experiment

The methodology used to obtain proteins with anti-tryptic activity was previously established and adapted by Medeiros et al. [29] to obtain pure TTI (TTIp). To obtain the flour and crude protein extract, tamarind seeds were grounded to a flour with fine granulation (40 mesh). The extraction was then done by adding 50 mM Tris-HCl buffer, pH 7.5 to the tamarind flour. This solution was kept under constant stirring at room temperature, and then the material was centrifuged and filtered to obtain the crude extract (EB). Subsequently, a protein fractionation with ammonium sulphate was performed in three saturation bands.

The protein fraction exhibiting the highest antitrypsin activity was subjected to a trypsin-Sepharose affinity chromatography (10 cm × 1.5 cm) in 50 mM Tris-HCl buffer, pH 7.5 for TTI isolation. Then, the column-adsorbed material was eluted with 5 mM HCl and collected in 3 mL aliquots at a flow rate of 0.5 mL/min.

The proteins adsorbed to the affinity column were dialysed against 50 mM Tris-HCl buffer, pH 7.5, lyophilized and designated TTI. TTI was analyzed and purified by reverse phase high performance liquid chromatography (HPLC) (Hilicon AB, Umea, Sweden), the proteins contained therein were analyzed and purified by reverse phase HPLC on Shimadzu LC-10A liquid chromatograph consisting of a binary solvent pumping system (LC-10ADvp) (Shimadzu Corporation, Kyoto, Japan), UV-Vis spectrophotometric detector (SPD-10A vp) (Mettler-Toledo Ind. e Com. Ltda., Barueri, São Paulo, Brazil), Rheodyne injector and workstation with SCL-10Avp system controller. The column used was the analytical column Vydac C18 218TP54 (Hichrom, Berkshire, England), 5 μm, 4.6 × 250 mm, 300 Å; solvent A was analytical grade water +0.1% TFA and solvent B was ACN/TFA 0.1%, with the ideal gradient for elution of the protein of interest. Elution of the protein content was monitored by UV detection at wavelengths 216 nm and 280 nm. The collections were performed manually and the protein peak of interest, denominated TTIp.

The trypsin inhibitor purified from tamarind seeds was termed TTIp. All purification steps were monitored by a trypsin inhibition assay using 1.25 mM BApNA (n-benzoyl-DL-arginine-p-nitroanilide) as a substrate according to Reference [30]. For the quantification of proteins, the Bradford method [31] was used with bovine serum (BSA) as the standard.

The material was frozen and stored at −20Â °C. Purification and molecular weight estimation of the various purification steps were evaluated by 12.5% denaturing and discontinuous polyacrylamide gel electrophoresis (SDS-PAGE) [32]. After electrophoresis, the proteins were stained with silver [33].

### 2.3. In Vivo Experiments

To evaluate the effect of the treatments (TTIp and standard diet), the experiments were performed with four groups of animals (*Wistar* rats, *n* = 5, male, adult). These groups were formed by animals fed an HGLI diet [34] and with nutritional diagnosis of obesity by the Lee Index ≥300 [35].

Initially, all 15 animals underwent 5 days of adaptation to establish the conditions of the experiment and the pattern of daily food intake. During the adaptation period, all animals received water by gavage and all procedures that occurred during the 10 days of experiments were performed and repeated daily.

The TTIp was administered at the concentration of 730 μg/kg per gavage/day. This concentration was defined as the result of the IC50 of TTIp [29], following the recommendations of Carvalho et al. [28].

All the animals, with the diagnosis of obesity and another eutrophic group fed a standard diet, were treated for 10 days, as described below:

Eutrophic/Standard diet (*n* = 5): received the Labina^®^ (Presence, Paulínia, São Paulo, Brazil) diet + 1 mL of water per gavage. This was the eutrophic control group, which did not receive any treatment;

Obese/HGLI diet (*n* = 5): received the experimental HGLI diet + 1 mL of water per gavage. This was considered the control obese group not receiving treatment;

Obese/Standard diet (*n* = 5): received the Labina^®^ diet + 1 mL of water per gavage. This was considered the obese group receiving conventional treatment; 

Obese/HGLI diet + TTIp (*n* = 5): received the experimental HGLI diet + 1 mL of TTIp (730 µg/kg) per gavage, considered the group in the test treatment. 

Animals had food consumption and their zoomometric measurements (weight, tail length, naso-anal length, and Lee index) evaluated on a daily basis. On the 11th day of the experiment, rats were anesthetized and sacrificed for visceral blood and fat collection and analysis of biochemical and molecular parameters.

### 2.4. Animals

Adult male Wistar rats were obtained from the Potiguar University (UnP) laboratory and randomly and individually distributed in polypropylene cages. Four groups of five animals were formed. Animals were kept under standard light conditions (12/12 h light/dark) and temperature (23–25 °C), water and food ad libitum. All experiments were performed in accordance with the Guide for the Care and Use of Laboratory Animals [36] and the study was approved by the Committee on Ethics in the Use of Animals (CEUA-UnP) under protocol of approval No. 012/2015.

### 2.5. Diets

Experimental diets offered to rats throughout the *in vivo* experiment were the Labina^®^, a standard and commercially available diet, and the HGLI diet. The HGLI diet contains 48% carbohydrates and has a glycemic index of 77.6 and a glycemic load of 38.8 [34,37] (Table 1).

### 2.6. Food Intake and Weight Gain Evolution

After the consumption pattern was established, these animals were treated with the diets described above and evaluated for changes in weight and food intake; 1 h after oral administration of the different diets. Prior to oral administration of the diets, the animals were fasted for a period of 6 h.

The results of food consumption (g) and weight (g) are expressed using the differences of the daily means during the treatments and demonstrated in percentage (%) according to the difference between the mean of the initial and final value of the consumption and weight gain, both in the adaptation period and at the end of each experiment.

### 2.7. Biochemical Parameters

On the last day of the experiment, animals were fasted for 8–12 h. Blood was then collected by the portal vein and sera used for the determination of fasting glycemia, triglyceride (TG), high density lipoproteins (HDL-C), low density lipoproteins (LDL-C), total cholesterol, and tumor necrosis factor-α (TNF-α). Animals were then euthanized in a CO_2_ chamber.

The method used for the biochemical parameters was enzymatic-colorimetric (Labtest^®^, Minas Gerais, Brazil). Serum samples were analyzed using commercially available immunoassay kits according to Vendrame et al. [38]. The quantikinerato TNF-α immunoassay kit (R&D Systems #RTA00, Minneapolis, MN, USA) was used. According to the manufacturer, the sensitivity of the kit was <15 pg/mL.

### 2.8. PPAR-γ and TNF-α Expression in Adipose Tissue 

Fats from the perirenal region (visceral) were removed by a longitudinal cut using surgical scissors from the base of the abdomen to the whole segment of the external showing the whole abdominal and thoracic cavities. 

mRNA of these tissues was extracted using TRIzol^®^ reagent (Thermo Fisher Scientific, Wilmington, DE, USA); 100 mg of fresh tissue was used in liquid nitrogen crucibles. Quantification of mRNAs was performed using an RNA assay kit (Invitrogen Life Technologies, Carlsbad, CA, USA). To assess the expression of *PPAR-γ* and *TNF*-α mRNA in adipose tissues, cDNA was prepared by reverse transcription of the total RNA. The cDNA was used for the polymerase chain reaction (PCR) containing TaqMan Universal PCR Master Mix for: Peroxisome proliferator-activated receptors (*PPAR-γ*, Rn00440945_m1) and Tumor Necrosis Factor Alpha (*TNF-α*, Rn01525859_g1), all of which are from Thermo Fisher Scientific—Wilmington, DE, USA. Assays were performed according to the manufacturer’s protocols. The relative expression was calculated using the 2−ΔΔCT method and the results are presented as changes compared to the mean values of the control group (eutrophic group), normalized for *GAPDH* mRNA, according to Livak and Schmittgen [39].

### 2.9. Immunohistochemistry of TNF-α

For immunohistochemistry, procedures were performed according to Khan et al. [40], with some modifications. The material was dehydrated and paraffin embedded, with sections of 3 μm thickness in a paraffin rotary microtome and subsequent assembly in gelatinized slides. These were then deparaffinized and hydrated. Antigen retrieval was performed using a heating plate for 20 minutes to reach 80 °C. Between each step, 5-minute wash cycles in 0.1 M sodium phosphate buffer, pH 7.4, were used.

For the detection of TNF-α in the adipose tissue, the Rabbit Specific HRP/DAB kit (ABC) (Abcam, Cambridge, UK) was used according to the manufacturer’s instructions, and the TNF-α rabbit polyclonal primary antibody (1:100 dilution) (Abcam, Cambridge, UK) was incubated overnight. For analysis, images of adipose tissue after immunohistochemistry were captured using a DS-Ri1 digital camera (Nikon, Edgewood, NY, USA) coupled to an Eclipse Ni (Nikon, Edgewood, NY, USA) (100×) microscope.

The intensity of TNF-α was measured semi-quantitatively and automatically by means of the determination of the optical density in diaminobenzidine staining (DAB) images since the optical density is proportional to the staining concentration. The evaluation was based on Image J software (version 1.51) (National Institutes of Health, Bethesda, Rockville, MD, USA) using a plugin known as IHC profiler [40]. 

The DAB staining images were analyzed pixel by pixel by this plugin and the scoring was given according to a grade, which consisted of the following variation: negative (0), low positive (+1), positive (+2), and high positive (+3). This method is validated and considered better and more reliable when compared to the qualitative method, in which there is only visual analysis [41].

### 2.10. Statistical Analysis

Sample size was calculated according to the 3Rs (Replacement, reduction, and refinement) principle, considering a simple and random sampling (Cochran model.) Also, according to the coefficient of variation, the difference between the treatments was considered significant. Thus, a physiologically significant difference of the parameters evaluated was assumed when the treatment (HGLI diet) exerted a biological effect of 25% or more in relation to the group that did not receive the diet. Also, the coefficient of anticipated variation of 10% was adopted, with an error probability of less than 5% and 90% power. The result was a “*n*” of 4.36 animals, which rounding up to the next whole number was five animals per group.

Data were analyzed for normality using the Kolmogorov-Smirnov test. All variables were considered non-parametric, and the Kruskal-Wallis test was used to verify if the weight gain, the daily consumption differences, and the biochemical data differed between the studied groups. When significant differences were detected, Dunn’s post-hoc test was used. Data were analyzed using Graph Pad Prism, version 5.0 (Graph Pad Software, San Diego, CA, USA).

## 3. Results

### 3.1. TTIp

The TTIp purification was confirmed by 12.5% SDS-PAGE (Figure S1), with a protein band of approximately 18 kDa, 40100.31 IU/mg of protein and 100% inhibition for trypsin.

### 3.2. Food Intake and Weight Gain

**Figure 1:**
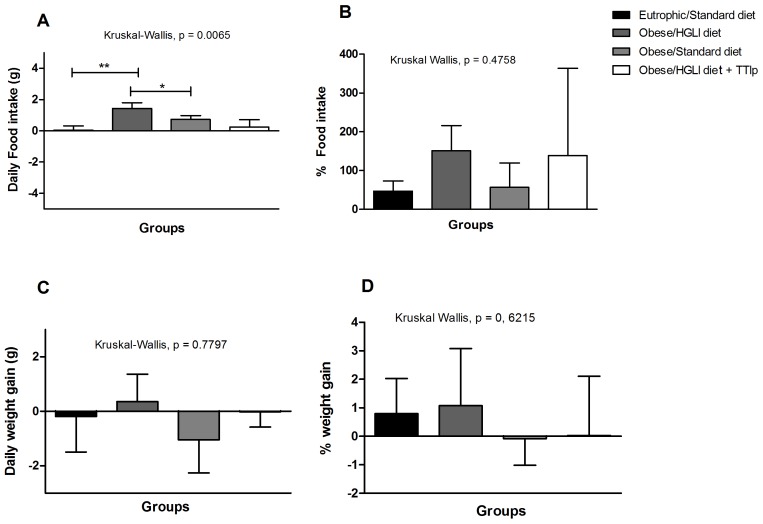
TTIp lowers on food intake but not weight gain in *Wistar* rats after 10 days of treatment. (**A**) Daily food intake (g). (**B**) Food intake percentage (%). (**C**) Daily weight gain (g). (**D**) Weight gain percentage (%). All treatments were performed for 10 days: Eutrophic/Standard diet (eutrophic animals receiving Labina^®^ diet + water by gavage), Obese/HGLI diet (obese animals receiving HGLI diet + 1 mL of water by gavage), Obese/Standard diet (obese animals receiving Labina^®^ diet + water by gavage), and Obese/HGLI diet + TTIp (obese animals receiving HGLI diet + 1 mL of TTIp by gavage at 730 μg/kg). All groups represent experiments with five animals. HGLI diet: Mixture composed of Labina^®^, condensed milk, and sugar (1: 1: 0.21); Standard diet: Labina^®^ chow. HGLI: High glycemic index and load diet. * Statistical difference between groups with the Kruskal-Wallis test, followed by Dunn’s post-hoc test, with *p* < 0.05. ** Statistical difference between Eutrophic/Standard diet and Obese/HGLI diet with the Kruskal-Wallis test, followed by Dunn’s post-hoc test, with *p* < 0.05 TTIp: Tamarind-purified trypsin inhibitor (*Tamarindus indica* L.).

Figure 1 shows the results of food intake and weight gain of obese animals treated with TTIp during the 10 days. The group treated with TTIp presented the lowest medians of food intake (Figure 1A), when compared to untreated animals (Kruskal-Wallis, *p* = 0.0065). Figure 1B shows the difference in dietary intake, considering the mean final and initial values in percentage (%), demonstrating that the group of rats that received only the HGLI diet presented higher food intake, followed by the group treated with TTIp, and the standard diet group (conventional treatment).

There was no statistical difference in weight gain (g) in the studied groups during the 10 days of the experiment (Figure 1C). However, total weight gain, considering the final and initial mean values, in percentage (%), was slightly higher in the group of animals without treatment, although not statistically different between the groups (Figure 1D).

### 3.3. Glucose 

**Figure 2:**
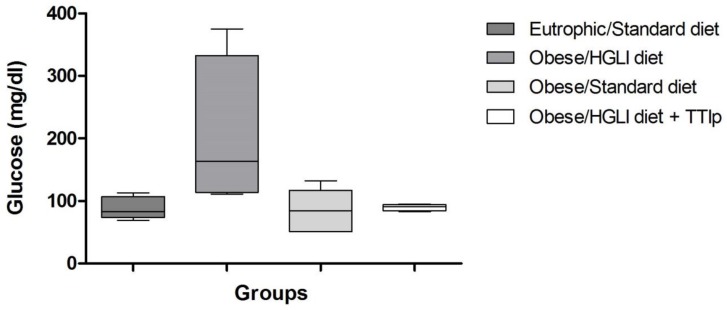
TTIp reduces fasting glycemia in *Wistar* rats after 10 days of treatment. Eutrophic/Standard diet (eutrophic animals receiving Labina^®^ diet + water by gavage). Obese/HGLI diet (obese animals receiving HGLI diet + 1 mL of water by gavage), Obese/Standard diet (obese animals receiving Labina^®^ diet + water by gavage), and Obese/HGLI diet + TTIp (obese animals receiving HGLI diet + 1 mL of TTIp by gavage at 730 μg/kg). All groups represent experiments with five animals. HGLI diet: Mixture composed of Labina^®^, condensed milk and sugar (1: 1: 0.21); Standard diet: Labina^®^ chow. HGLI: High glycemic index and load diet. TTIp: Tamarind-purified trypsin inhibitor (*Tamarindus indica* L.).

According to Figure 2, animals treated with TTIp and the standard diet reduced fasting glycemia when compared to the group of untreated obese rats, which kept consuming the HGLI diet. In addition, this Figure also shows the dispersion/homogeneity of the data, demonstrating that the TTIp treated group presented less dispersed and more homogeneous results.

### 3.4. Lipid Profile

**Figure 3:**
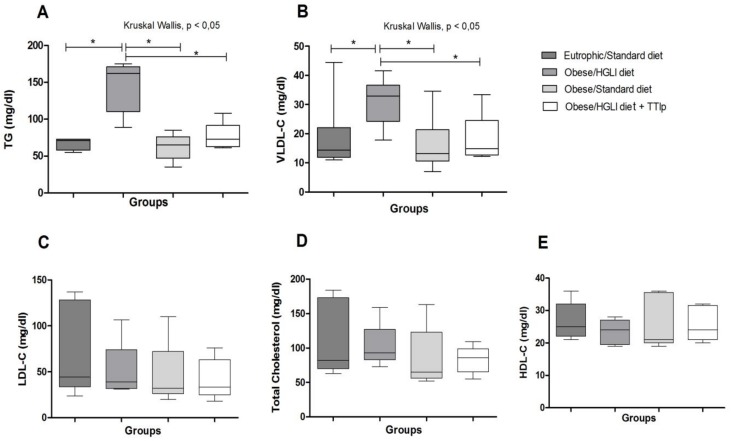
Effect of TTIp on lipid profile of *Wistar* rats after 10 days of treatment. (**A**) TG: Triglycerides. (**B**) VLDL-C: very low density lipoprotein. (**C**) LDL-C: low density lipoprotein. (**D**) Total Cholesterol. (**E**) HDL-C: high-density lipoprotein. Eutrophic/Standard diet (eutrophic animals receiving Labina^®^ diet + water by gavage). Obese/HGLI diet (obese animals receiving HGLI diet + 1 mL of water by gavage), Obese/Standard diet (obese animals receiving Labina^®^ diet + water by gavage), and Obese/HGLI diet + TTIp (obese animals receiving HGLI diet + 1 mL of TTIp by gavage at 730 μg/kg). All groups represent experiments with five animals. HGLI diet: Mixture composed of Labina^®^, condensed milk and sugar (1: 1: 0.21); Standard diet: Labina^®^ chow. HGLI: High glycemic index and load diet. TTIp: Tamarind-purified trypsin inhibitor (*Tamarindus indica* L.). * Statistical difference between groups with the Kruskal-Wallis test, followed by Dunn’s post-hoc test, with *p* < 0.05.

Obese groups treated with TTIp and standard diet presented the lowest TG concentrations (76.20 ± 18.73 and 62.2 ± 18.0 mg/dL) and VLDL-C (15.24 ± 3.75 and 12.4 ± 3.6 mg/dL), respectively (Figure 3A,B), when compared to the obese group receiving the HGLI diet (Kruskal-Wallis *p* = 0.0108). Concerning TG concentrations, the TTIp-treated group presented less dispersed and more homogeneous results.

### 3.5. TNF-α

All of the obese animals treated with TTIp presented undetectable levels of TNF-α (Table 2), similarly to the eutrophic group receiving the standard diet (healthy group). This was not observed in the other obese studied groups. 

**Figure 4:**
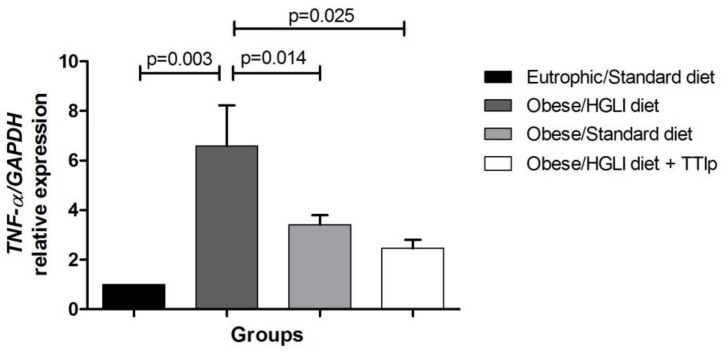
TTIp reduces relative *TNF-α* mRNA expression in perirenal adipose tissue in *Wistar* rats after 10 days of treatment. Eutrophic/Standard diet (eutrophic animals receiving Labina^®^ diet + water by gavage). Obese/HGLI diet (obese animals receiving HGLI diet + 1 mL of water by gavage), Obese/Standard diet (obese animals receiving Labina^®^ diet + water by gavage), and Obese/HGLI diet + TTIp (obese animals receiving HGLI diet + 1 mL of TTIp by gavage at 730 μg/kg). All groups represent experiments with five animals. HGLI diet: Mixture composed of Labina^®^, condensed milk and sugar (1: 1: 0.21); Standard diet: Labina^®^ chow. HGLI: High glycemic index and load diet. TTIp: Tamarind-purified trypsin inhibitor (*Tamarindus indica* L.). *GAPDH*: Glyceraldehyde-3-phosphate Dehydrogenase (Rn01775763_g1). *TNF-α:* tumor necrosis fator-α (LOC103694, Rn01525859_g1). Statistical difference between groups, with the Mann-Whitney test and *p* < 0.05.

The relative expression of *TNF-α* mRNA in perirenal adipose tissue was lower in obese animals treated with the standard diet (*p* = 0.014) and TTIp (*p* = 0.025) when compared to the untreated obese group (Figure 4).

### 3.6. Immunohistochemistry of TNF-α

**Figure 5:**
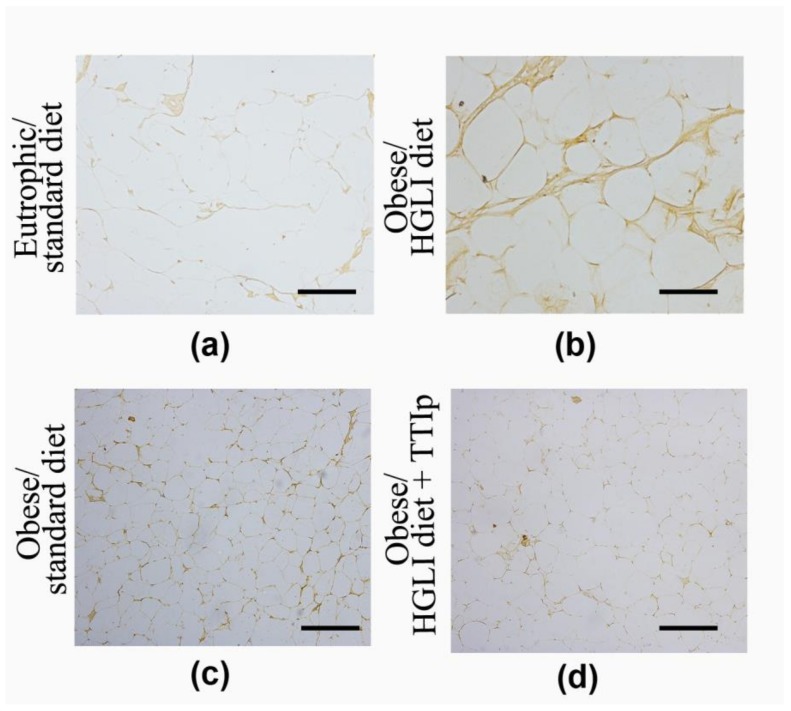
Effect of TTIp on immunostaining of TNF-α in adipose tissue of *Wistar* rats after 10 days of treatment. All groups represent experiments with *n* = 5 animals. (**a**) Perirenal adipocytes in the eutrophic group/standard diet; (**b**) Perirenal adipocytes in the obese group/HGLI diet; (**c**) Perirenal adipocytes in the obese group/standard diet; (**d**) Perirenal adipocytes in the obese group/HGLI diet + TTIp. Bars indicate 1000 µm. HGLI diet: Mixture composed of Labina^®^, condensed milk and sugar (1: 1: 0.2); TTIp diet: *Tamarindus indica* L. seed purified trypsin inhibitor.

Immunohistochemistry showed a discrete immunostaining of TNF-α in the adipose tissue of obese animals treated with TTIp and control animals, unlike the HGLI group, which presented intense staining of this cytokine in perirenal tissue compartments evaluated (Figure 5) with negative immunostaining (0) by optical density (Table 3).

### 3.7. PPAR-γ

**Figure 6:**
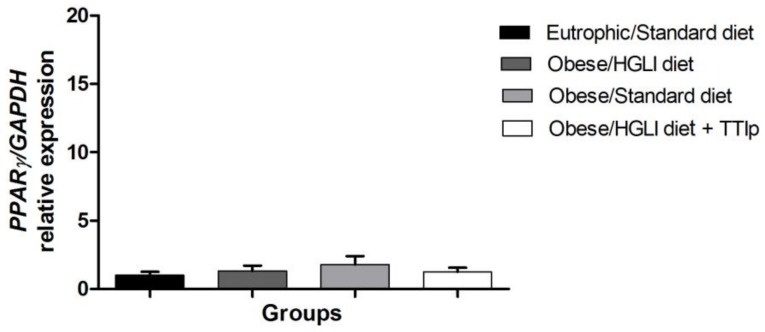
TTIp presented no effect on relative *PPAR-γ* mRNA expression in perirenal adipose tissue in *Wistar* rats after 10 days of treatment. Eutrophic/Standard diet (eutrophic animals receiving Labina^®^ diet + water by gavage). Obese/HGLI diet (obese animals receiving HGLI diet + 1 mL of water by gavage), Obese/Standard diet (obese animals receiving Labina^®^ diet + water by gavage), and Obese/HGLI diet + TTIp (obese animals receiving HGLI diet + 1 mL of TTIp by gavage at 730 μg/kg). All groups represent experiments with five animals. HGLI diet: Mixture composed of Labina^®^, condensed milk and sugar (1: 1: 0.21); Standard diet: Labina^®^ chow. HGLI: High glycemic index and load diet. TTIp: Tamarind-purified trypsin inhibitor (*Tamarindus indica* L.). *GAPDH*: Glyceraldehyde-3-phosphate dehydrogenase (Rn01775763_g1). *PPAR-γ*: Peroxisome proliferator-activated receptors.

There was no difference between the studied groups for the relative expression of *PPAR-γ* mRNA in the perirenal adipose tissues (Figure 6).

## 4. Discussion 

In previous studies, Medeiros et al. [29] purified TTIp (tamarind Kunitz trypsin inhibitor). In this study, TTIp was again obtained, as visualized by SDS-PAGE. Its satietyogenic effect on biochemical and molecular variables was evaluated in *Wistar* rats with obesity and dyslipidemia. 

In the present study, in obese animals, TTIp exerted its satietyogenic effect similarly to when it was partially purified [28]. The process of purifying a protein can directly influence its bioactivity. Thus, the partially purified protein complex (TTI) when fully purified (TTIp) could have lost the bioactivities previously presented [28,29,42], since these activities in proteins are directly related to their structures and conformations [43].

In the study by Lima et al. [44], the protein isolate of *Erythrina velutina* seeds showed high activity for human neutrophilic elastase (HNE), a characteristic that was not observed by Machado et al. [45], when purifying the protein isolate of *Erythrina velutina* (EVTI) using high performance liquid chromatography (HPLC) after the affinity column Trypsin-Sepharose CNBr 4B. In the study, EVTI showed high inhibitory efficiency on trypsin and did not inhibit HNE. 

In the present study, obese animals presented lower food consumption when treated with TTIp, compared to the other evaluated groups. It is noteworthy that from the four groups, two continued to consume during the 10 days of experiment the same diet that was able to induce obesity and only one group started to consume the standard diet (nutritionally adequate). Thus, in this case, because they adhered to a nutritionally adequate diet, animals with obesity reduced their consumption, similarly to the effect caused by the use of TTIp.

Regarding weight gain, consistency was observed between the results obtained with food consumption, but there was no significant statistical difference between the groups. However, there was an increase in weight gain in the group that consumed the HGLI diet without treatment. In addition, a significant reduction of weight gain was observed in the groups of animals treated with TTIp and standard diet.

The reduction in dietary intake and the consequent weight loss observed in studies with trypsin inhibitors are more common in eutrophic animals, although they have been administered in a short period of time and are mainly attributed to the secretagogue effect and consequent serum increase of CCK [23,46,47,48]. However, it is seen that in obese rodent, the effect on the satiety of this hormone in comparison to eutrophic animals occurs independently of the serum concentration of CCK when stimulated by different secretagogues [42,49,50].

In the study by Costa et al. [42], with obese *Wistar* rats, the reduction in dietary intake due to TTI (25 mg/kg) administered during 10 days was regardless of the increase of serum CCK. This reduction may have been caused by a significant decrease in the plasma concentration of leptin in these animals, an effect also attributed to TTI [42], since leptin directly influences the satietyogenic effect of CCK [51,52,53].

Medeiros et al. [29] in a study with TTIp (730 μg/kg) found that the same effect of leptin reduction was observed in obese rats, without action on CCK, also described by Costa et al. [42] using the same molecule, still partially purified. Therefore, the satietyogenic effect presented by TTIp in this study can also be justified by the improvement of the sensitivity to CCK, regardless of its plasma increase, which is conditioned by the reduction of leptin, which is usually high in obesity. However, leptin was reduced in obese animals treated with TTIp [29].

Furthermore, enzymatic inhibitors act by regulating not only hormones, but also several biochemical parameters. Serquiz et al. [48], in his study on the effect of the isolated inhibitor of peanut (AHTI) on eutrophic animals, revealed its action in the reduction of fasting glycemia. Analyzing the effects of TTIp on biochemical parameters, there was a significant difference between the studied groups regarding the decrease on plasma concentrations of VLDL-C and TG, in which the group treated with TTIp and the standard diet presented lower concentrations than the groups of untreated obese rats.

In the group of animals in which the standard diet was administered, the reduction in lipid parameters (VLDL-C and TG) is probably due to the fact that the diet has normoglycemic and normolipid characteristics, different from the HGLI diet with high glycemic load and high sugar concentrations. *Wistar* rats submitted to diets with high glycemic index (45% of the total energetic value of the diet derived from carbohydrates, 20% of proteins and 35% of lipids) showed significantly increased basal triglyceridemia when compared to rats fed the diet containing the same percentage composition of macronutrients, but with low glycemic index carbohydrates [54].

The acute intake of carbohydrates promotes its own oxidation, reducing fat oxidation, which is deposited, increasing body adiposity [55,56]. This effect is higher when carbohydrates have a high glycemic index. For example, in a 32-week study, animals submitted to diets based on high glycemic index carbohydrates presented higher weight gain, higher increase in visceral adiposity, and higher concentrations of lipogenic enzymes than animals submitted to diets with the same content energy, but with a lower glycemic index [57].

In addition, this characteristic of the HGLI diet may justify, for example, the mean absolute values of glycemia, VLDL-C and TG in animals that were kept on the HGLI diet without treatment throughout the experiment; as well as the inflammatory status found, with high concentrations of TNF-α, besides obesity itself.

There are countless studies suggesting that hyperglycemic diets can increase triglyceridemia, favor the formation of small and dense LDL-C particles, and reduce plasma HDL-C concentrations [58,59,60]. Two possible mechanisms have been identified as responsible for the increase in triglycerideemia induced by carbohydrates: (1) increased triglyceride synthesis and consequent increased production and release of VLDL by the liver and (2) decreased clearance of plasma TG-rich particles [61]. 

Hyperlipidic and hyperglycemic diets considerably stimulate lipogenesis [1], increasing the expression of lipogenic enzymes [2]. The stimulation of lipogenic enzymes can occur through transcription factors such as SREBP3 and ChREBP, which is activated in response to high glycemia [1] and stimulation of the nuclear receptor PPAR-γ [4]. Glucose itself, because of the conversion to acetyl-CoA in the glycolytic pathway, stimulates lipogenesis, which is a substrate for this process. In addition, plasma glucose stimulates lipogenesis, acting on the insulin release process. Insulin is probably the most important hormonal factor affecting lipogenesis, stimulating it in a very potent way, increasing the uptake of glucose by adipose cells, by recruiting glucose transporters to the plasma membrane, as well as activating glycolytic and lipogenic enzymes [4].

Mittendorfer and Sidossis [62] submitted healthy individuals to a hyperglycemic diet (75% of energy in the form of carbohydrates and 10% in the form of fats) and to a hyperlipid diet (30% carbohydrate and 55% fat), and analyzed the metabolism of VLDL from isotopically labeled VLDL particles. It was concluded that the highest concentration of VLDL occurred after the hyperglycemic diet. The possible explanation for the higher secretion of VLDL was that it would be a result of the lower oxidation of plasma-derived fatty acids as observed in the study, after the consumption of the hyperglycemic diet, which caused an increase in the availability of fatty acids for the hepatic synthesis of triglycerides.

Interestingly, TTIp also caused the reduction of VLDL-C and TG concentrations in obese animals that remained in the HGLI diet. We also observed that blood glucose values of obese animals in the HGLI diet were similar to those of the eutrophic animals and to those of the obese animals treated with the nutritionally adequate diet. TTIp may be acting in some of these pathways, reducing the expression of lipogenic enzymes, or even reducing the expression of transcription factors such as SREBP and ChREBP. Since it did not influence the expression of the *PPAR-γ* gene, it is a candidate for the induction of lipogenesis conditioned by high glycemic index diets [28,63,64].

PPARγ regulates the expression of numerous genes involved in lipid metabolism, including Adipocyte protein 2 [65], acyl-CoA synthetase [66] and LPL [67]. It also controls the expression of the fatty acid carrier protein 1 and cluster of differentiation 36, both of which are involved in lipid uptake by adipocytes [66]. PPARγ has a regulatory role in adipogenesis [68,69]. A recent study suggested that PPARγ activation may decrease the progression of atherosclerosis and increase insulin sensitivity, and may be a potential therapeutic target for the treatment of various diseases, including type 2 diabetes mellitus and dyslipidemia [70].

In the literature, there is also the classic example of statins, which are drugs that act to reduce dyslipidemia and have competitive kinetics, competing reversibly with 3-hydroxy-3-methyl-glutaryl-coenzyme A (HMG-CoA) for an active site of the enzyme HMG-CoA reductase, inhibiting the synthesis of cholesterol [71]. Accordingly, TTIp is a competitive inhibitor for trypsin [29]. Thus, its performance may be similar to statins, which may not only act on the expression of genes involved in this reversal, but also by reverse or irreversible competition with the lipogenic enzymes. This may be of great relevance to the pharmaceutical industry, since inhibitors with this characteristic are mostly non-competitive [72].

In this study, when TNF-α was analyzed as a marker of inflammation, it was reduced to undetectable concentrations in all animals treated with TTIp. This is an interesting result, since TNF-α plays a central role in chronic inflammation [73,74,75]. In addition, considering that TNF-α concentrations are undetectable in healthy humans and animals [76,77], it can be stated that TTIp had a significant effect on the reduction of inflammation in obese animals, reducing this inflammation to concentrations equal to those of healthy animals, regardless of weight loss. It is noteworthy that in our study, eutrophic animals also presented undetectable TNF-α concentrations.

Corroborating with what was previously mentioned, in obese animals treated with TTIp, a significant decrease in the relative expression of *TNF-α* mRNA tissue was observed with negative immunostaining in the visceral adipose. Studies show that TNF-α is a cytokine that acts directly on the adipocyte, promoting the induction of apoptosis, inhibition of lipogenesis, and inhibition of LPL expression of GLUT-4 and acyl-CoA synthetase, thus fulfilling an important regulatory role in the accumulation of fat in adipose tissue [74,75,76,78]. Thus, all animals with obesity were expected to show high concentrations of both TNF-α plasma concentrations, *TNF-α* gene expression and immunostaining for TNF-α positive in the analyzed adipose tissue.

In the acute phase of inflammation, increased production of TNF-α and interleukins such as IL-6 and IL-1β are characteristic. TNF-α, IL-6, and IL-1β are secreted by macrophages and have systemic and local effects. In addition, its increased expression appears to be related to the reduction of insulin receptor substrate-1 (IRS-1), GLUT-4 and adiponectin expression in adipose tissue, and stimulation of the production of CRP and more IL-6 [69,74]. TNF is responsible for the activation of endothelial cells, stimulating the exposure of integrins and selectins that participate in the leukocyte rolling process [79].

TNF-α is at the top of the inflammatory cascade and is responsible for the activation of lymphocytes, stimulation of the release of proteolytic enzymes by macrophages, and production of other inflammatory cytokines such as IL-13. Thus, cytokines play a key role in the perpetuation of inflammation, through feedbacks that control immune activity, which are present in rheumatoid arthritis, ankylosing spondylitis, obesity, atherosclerosis, inflammatory bowel diseases, metabolic syndrome, and type 2 diabetes [17,19,80,81].

For the treatment of some pathologies, such as rheumatoid arthritis, some types of drugs such as disease-modifying drugs (DMARDs) are commonly used [82,83,84]. This group of drugs presents a series of chemically unrelated drugs, such as antimalarial drugs, methotrexate, leflunomide, IL-1 receptor antagonists, cytotoxic agents, and biological TNF-α blocking agents [83,85,86].

Among the biological agents approved for the treatment of rheumatoid arthritis are those that act as TNF-α antagonists, called anti-TNF-α [17,18]. Currently, three agents that block the action of TNF-α are available in Brazil: etanercept, infliximab, and adalimumab [19,20]. However, all of these medications have adverse effects, increasing triglycerides, as well as the onset of type 2 diabetes, in addition to the risk of atherosclerosis [20].

It is worth noting that in this study, TTIp acted in a way to reduce both the serum TNF-α to undetectable concentrations and the relative expression of its gene with negative immunostaining in the visceral adipose tissue. These findings demonstrate that it can act as a potent anti-TNF-α, acting as a blocker or even TNF-α antagonist. Moreover, TTIp not only reduced inflammation, but also reduced the serum VLDL-C and TG. Thus, TTIp may also decrease cardiovascular risk as well as the lipogenic process of obesity. This is of great relevance to the pharmaceutical industry; once up to date, there are no known anti-TNF-α pharmaceuticals that do not alter the lipid profile. 

## 5. Conclusions

Taken altogether, our results allow us to conclude that obese and dyslipidemic animals when treated with TTIp reduced food intake and chronic inflammation, by reducing serum TNF-α concentrations and the relative expression of its gene in adipose visceral tissue. In addition, TTIp also acted, reducing the dyslipidemia presented by the animals with obesity induced by a diet of high glycemic index and load regardless of *PPAR-γ* induction.

## Figures and Tables

**Table 1:** Centesimal composition (g/100 g) of experimental and standard diets.

		Diets	
	Experimental	Standard	Labina’s^®^ Label
Kcal (Calories)	315.26 ± 19.27 (100%)	265.45 ± 7.99 (100%)	-
Moisture	4.54 ± 0.03 (5%)	7.47 ± 0.30 (7%)	Maximum: 13.0%
Ashes	9.22 ± 0.09 (9%)	9.84 ± 0.04 (10%)	-
Proteins	20.72 ± 1.08 (21%)	28.59 ± 0.25 (29%)	Minimum: 23.0%
Lipids	4.34 ± 0.17 (4%)	3.74 ± 0.06 (4%)	Minimum: 2.5%
Carbohydrates *	48.33 ± 5.38 (48%)	29.35 ± 2.08 (29%)	-
Crude fiber	12.85 ± 4.68 (13%)	21.00 ± 1.68 (21%)	Maximum: 9.0%

Experimental diet: Mixture of Labina^®^, condensed milk and sugar (1:1:0.21); Standard diet: Labina^®^. * Calculated by difference (100 g of food—total sum of values found for moisture, lipids, ashes, proteins and fibers).

**Table 2:** Effect of TTIp on TNF-α in *Wistar* rats submitted to different treatments for 10 days.

Parameter	Eutrophic/Standard Diet	Obese/HGLI Diet	Obese/Standard Diet	Obese/HGLI Diet + TTIp
*TNF-alpha*	Indetectable	5.86 ± 0.43	6.53 ± 0.96	Indetectable

Eutrophic/Standard diet (eutrophic animals receiving Labina^®^ diet + water by gavage). Obese/HGLI diet (obese animals receiving HGLI diet + 1 mL of water by gavage), Obese/Standard diet (obese animals receiving Labina^®^ diet + water by gavage), and Obese/HGLI diet + TTIp (obese animals receiving HGLI diet + 1 mL of TTIp by gavage at 730 μg/kg). All groups represent experiments with five animals. HGLI diet: Mixture composed of Labina^®^, condensed milk and sugar (1: 1: 0.21); Standard diet: Labina^®^ chow. HGLI: High glycemic index and load diet. TTIp: Tamarind-purified trypsin inhibitor (*Tamarindus indica* L.).

**Table 3:** Score of TNF-α immunostaining in adipose tissue of *Wistar* rats submitted to different treatments during 17 weeks.

	Eutrophic/Standard diet ^a^	Obese/HGLI diet ^b^	Obese/Standard diet	Obese/HGLI + TTIp diet ^c^
**Negative (0)**	4	-	-	5
**Low positive (+1)**	1	1	3	-
**Positive (+2)**	-	4	1	-
**High positive (+3)**	-	-	1	-

All groups represent experiments with *n* = 5 animals. TNF-α: Necrosis Factor-Alpha. ^a^ Standard diet: Labina^®^ diet. ^b^ HGLI diet: Mixture composed of Labina^®^, condensed milk and sugar (1: 1: 0.2). ^c^ TTIp: Tamarind-purified trypsin inhibitor (*Tamarindus indica* L.).

## Supplementary Materials

The following are available online at https://www.mdpi.com/2072-6643/11/3/512/s1, Figure S1: 12.5% SDS-Polyacrylamide Gel Electrophoresis stained with silver nitrate of purification steps of Tamarind seed Inhibitor Trypsin.
