# Supplementary material for: Anti-TNF-α Agent Tamarind Kunitz Trypsin Inhibitor Improves Lipid Profile of Wistar Rats Presenting Dyslipidemia and Diet-induced Obesity Regardless of PPAR-γ Induction
Source: Nutrients. 2019 Feb 27;11(3):512. doi: 10.3390/nu11030512 (PMC6470745; doi:10.3390/nu11030512)
Supplement: Supplementary file 1 [file nutrients-11-00512-s001.pdf]

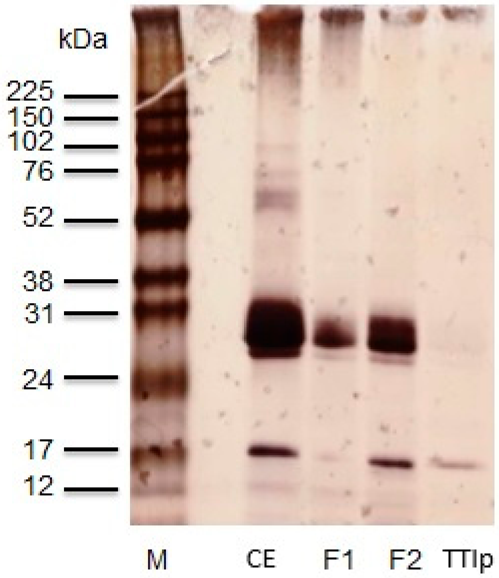

Figure S1. 12.5% SDS-Polyacrylamide Gel Electrophoresis stained with silver nitrate of purification steps of Tamarind seed Inhibitor Trypsin. M: Marker (São Paulo, Brazil) (Rainbow™); CE: Crude Extract (15μg); F1: fraction 0%–30% of ammonium sulfate (15μg); F2: fraction F2 saturated with 30%–60% of ammonium sulfate (15μg); ITTp: Purified Tamarind Seed Trypsin Inhibitor (TTIp) (17μg).
